# Case Report: Endovascular repair of thoracoabdominal aneurysm aorta and generalized mycobacterial infection (clinical case)

**DOI:** 10.3389/fcvm.2025.1623403

**Published:** 2025-11-06

**Authors:** Mikhail Chernyavskiy, Almaz Vanyurkin, Ekaterina Verkhovskaya, Yuliya Panteleeva, Daria Ryzhkova, Ilona Basek, Anton Ryzhkov, Lubov Mitrofanova, Dmitry Gulyaev, Galina Salogub, Ivan Danilov, Dmitry Ovchinnikov, Anna Starshinova, Dmitry Kudlay, Evgeny Shlyakhto

**Affiliations:** 1 Department of Vascular and Endovascular Surgery, Almazov National Medical Research Centre, Saint Petersburg, Russia; 2 Research Department, Nuclear Medicine and Teranostics, Institute of Oncology and Hematology, Almazov National Medical Research Centre, Saint Petersburg, Russia; 3 Department of Nuclear Medicine and Radiation Technologies, Clinic of the Institute of Medical Education, Almazov National Medical Research Centre, Saint Petersburg, Russia; 4 Department of Radiation Diagnostics, Almazov National Medical Research Centre, Saint Petersburg, Russia; 5 Department of Magnetic Resonance Imaging, Almazov National Medical Research Centre, Saint Petersburg, Russia; 6 Department of Pathomorphology, NIL Pathomorphology, Almazov National Medical Research Centre, Saint Petersburg, Russia; 7 Laboratory of Integrative Neurosurgical Technologies, Almazov National Medical Research Centre, Saint Petersburg, Russia; 8 Institute of Oncology and Hematology, Almazov National Medical Research Centre, Saint Petersburg, Russia; 9 Department of Faculty Surgery, Clinic of the Medical Faculty, Institute of Medical Education, Almazov National Medical Research Centre, Saint Petersburg, Russia; 10 University Clinic, Almazov National Medical Research Centre, Saint Petersburg, Russia; 11 Research Department, Almazov National Medical Research Centre, Saint Petersburg, Russia; 12 Laboratory of Probabilistic Methods in Analysis, Faculty of Mathematics and Computer Science, Saint Petersburg State University, Saint Petersburg, Russia; 13 Department of Pharmacology, Institute of Pharmacy, I.M. Sechenov First Moscow State Medical University, Moscow, Russia; 14 Laboratory of Personalized Medicine and Molecular Immunology, Institute of Immunology FMBA of Russia, Moscow, Russia; 15 Department of Pharmacognosy and Industrial Pharmacy, Faculty of Fundamental Medicine, Lomonosov Moscow State University, Moscow, Russia; 16 Almazov National Medical Research Centre, Saint Petersburg, Russia

**Keywords:** aortic aneurysm, aortic tuberculosis, aortic endovascular repair, mycobacterial infection, psoas abscess, spondylitis

## Abstract

Mycotic aortic aneurysms account for only 0.7%–1.3% of all aortic aneurysms but remain a life-threatening vascular complication, with in-hospital mortality rates reaching 36%. Immediate surgical intervention is required due to the high risk of rupture. Standard management combines targeted antimicrobial therapy with a radical resection of infected tissues and vascular reconstruction, yet the choice of optimal surgical strategy in elderly, comorbid patients remains controversial. Endovascular interventions are pivotal in the treatment of patients at high risk of infections, offering a minimally invasive approach that reduces the likelihood of complications and improves treatment outcomes. In this study, we report the case of a 65-year-old man who presented with severe lumbar pain. He was diagnosed with mycobacterial spondylitis of the L2–L4 vertebrae and a right-sided psoas abscess secondary to a ruptured thoracoabdominal mycotic aneurysm of 6-month duration. For diagnostic purposes, a right retroperitoneal mass was excised, and a histological examination demonstrated features consistent with mycobacterial infection. Based on these findings, antituberculosis chemotherapy was initiated. Considering the patient's advanced age and comorbidities, branched endovascular aortic repair (BEVAR) was performed as a less invasive alternative to open surgery. This rare clinical case demonstrated the feasibility of BEVAR as a therapeutic option for a ruptured thoracoabdominal aortic aneurysm complicated by disseminated mycobacterial infection and psoas abscess. The successful outcome suggested that BEVAR could serve as an effective alternative to open repair in selected high-risk patients.

## Introduction

Mycobacteriosis is a disease affecting the lungs and other organs caused by atypical (non-tuberculous) mycobacteria, which differ from the *Mycobacterium tuberculosis complex* in terms of their faster growth on nutrient media, ability to form pigments, and activity of certain enzymes, which are taken into account in their classification (1, 2). *Non-tuberculous mycobacteria* (NTM) are those found in dust, soil, and water (3). In recent years, interest in mycobacterial infections has increased significantly because of the large number of patients with mycobacterial lesions in various organs. Current projections indicate that the number of NTM infections will triple by the year 2040 (2). The most common pathogens in humans are the *Mycobacterium avium complex* (MAC), consisting of *Mycobacterium avium* and *Mycobacterium intracellulare*, and *Mycobacterium abscessus* (MAB) (4). With improvements in laboratory diagnostic capabilities, cases of mycobacterial infection are being reported with increasing frequency (5). However, it is difficult to obtain accurate data on the prevalence of NTM infection because it is not a reportable disease in all countries. Data from Queensland, Australia, where NTMs are a reportable disease, indicate that the number of NTM cases has more than doubled in the past few years, from 672 cases in 2012 (6) to 1,490 cases in 2022 (2).

Mycobacterial aortic aneurysms represent only 0.7%–1.3% of all aortic aneurysms. Nevertheless, they represent a life-threatening vascular complication associated with an in-hospital mortality of up to 36% (7).

It should be noted that non-tuberculous mycobacteria, as well as *M. tuberculosis complex*, are often resistant to first-line antituberculosis drugs and most other antibacterial agents (3). In countries with a high prevalence of tuberculosis (TB) infection, the problem of differential diagnosis with NTM remains difficult and urgent. TB remains one of the major infectious diseases that most often lead to death worldwide.

Endovascular interventions are pivotal in the treatment of patients at high risk of infections, offering a minimally invasive approach that reduces the likelihood of complications and improves treatment outcomes. This method is particularly important in patients with complex vascular pathologies, in whom the application of conventional surgical methods carries an increased risk of infectious complications.

Here, we discuss the clinical presentation, diagnostic approach, and treatment options for tuberculous thoracoabdominal aortic aneurysms, emphasizing the utility of endovascular repair.

## Clinical case

**Figure 1:**
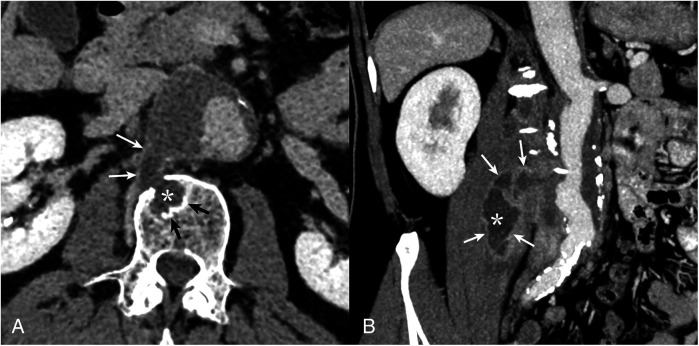
CT angiography of the abdominal aorta. Multiplanar CT reconstructions demonstrate communication of the contents of the vertebral body defect (**A**), with the cavity of the m. psoas major abscess (**B**) and the aortic lumen. Borderline (contact) sclerotic changes in the vertebral bodies (black arrows) and late (delayed) accumulation of contrast in the abscess capsule (white arrows) characteristic of the infectious process (asterisk) are noteworthy.

A 65-year-old man was admitted to the vascular surgery department of the Almazov Centre in March 2024 with severe lumbar pain. He had a known thoracoabdominal aortic aneurysm (30 mm since 2020), which had expanded to 50 mm with a para-aortic mass extending to the right m. psoas and right diaphragmatic pedicle, suggesting rupture (Figure 1).

### Diagnostic journey and key findings

**Figure 2:**
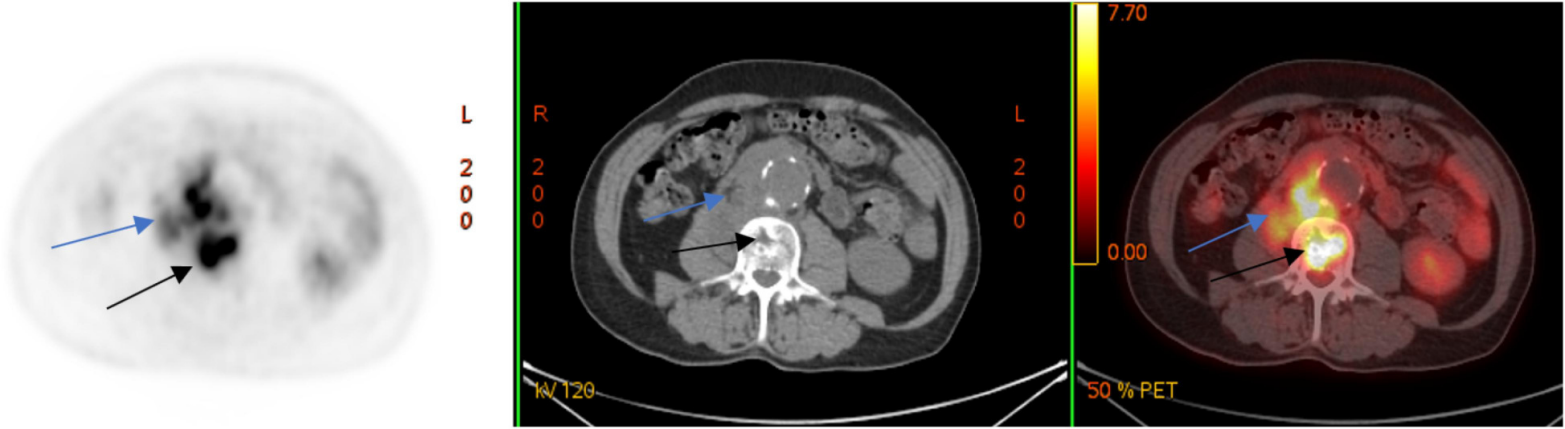
^18^F-FDG PET/CT (axial slices) demonstrates the presence of a metabolically active retroperitoneal infiltrate with extension to the right m. psoas (blue arrows) and lumbar vertebral bodies (black arrows).

A laboratory examination on 12 March 2024 showed elevated levels of C-reactive protein (CRP, 26.62 mg/L) and leukocytes 6.4 × 10^9^/L. To clarify the diagnosis, a couple of days later, the patient underwent positron emission tomography combined with computed tomography (PET/CT) with the use of ^18^F-fluorodeoxyglucose (^18^F-FDG), which revealed a metabolically active retroperitoneal infiltrate extending to the right psoas muscle and the L2–L3 vertebral bodies (Figure 2).

The initial differential diagnosis included malignancy, IgG4-related myofibroblastic tumor, retroperitoneal IgG4-associated fibrosis, non-Langerhans cell histiocytosis (Erdheim–Chester disease), and infection. An MRI demonstrated a ruptured thoracoabdominal aortic aneurysm (TAAA) with extension into the L2–L3 vertebral bodies and psoas muscle, resulting in vertebral body destruction (spondylitis) and a multicystic lesion in the right psoas (lysed hematoma). Serum IgG4 levels were within the normal range, supporting an infectious etiology.

To verify the pathogen and guide antimicrobial therapy, a vertebral biopsy was performed, which showed osteomyelitis without microbial growth. An empirical prolonged combined antimicrobial therapy was initiated with 600 mg of clindamycin administered every 8 h intravenously and 1.2 g of amoxicillin/clavulanate administered every 8 h intravenously.

**Figure 3:**
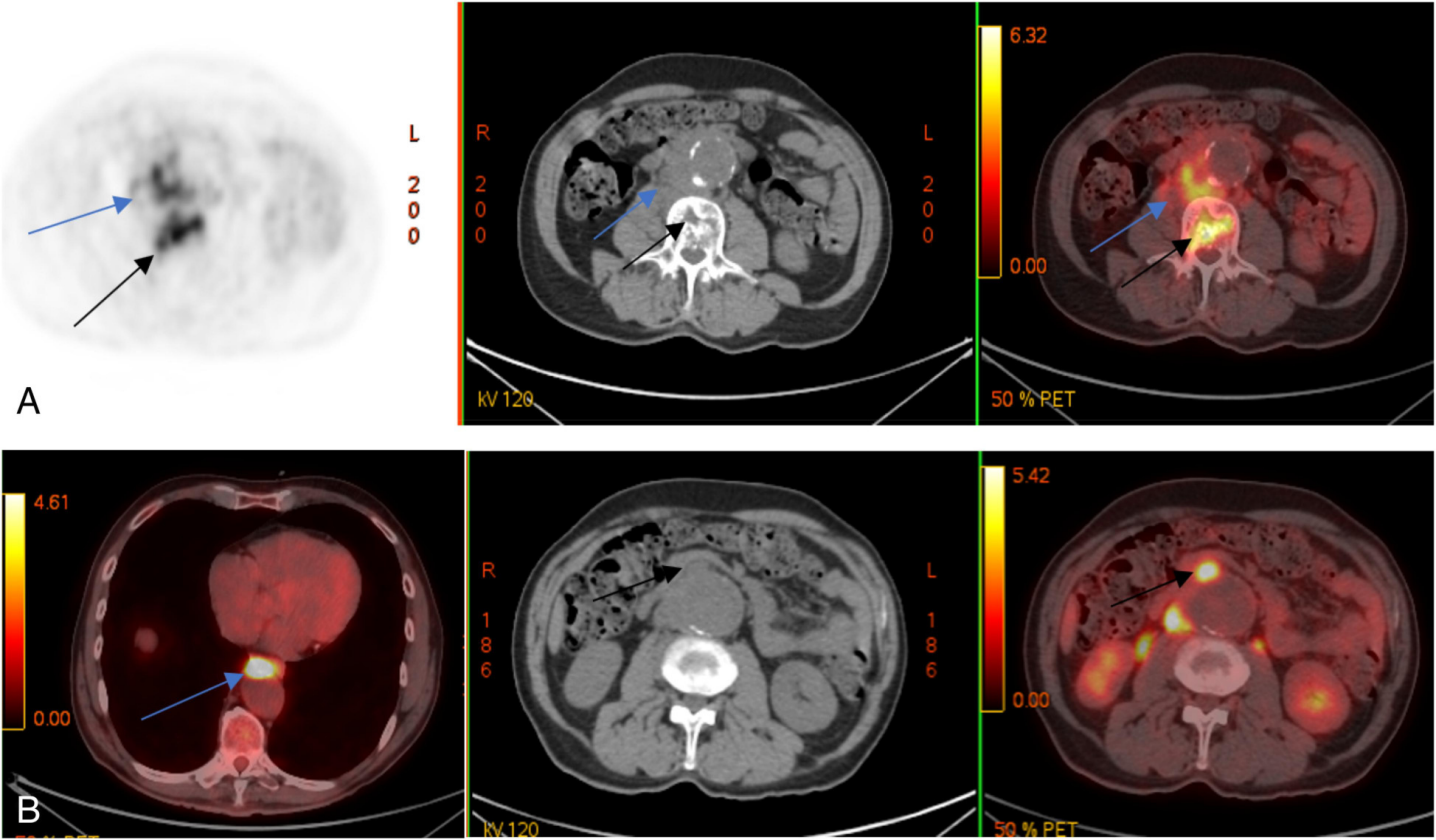
Control PET/CT with 18F-FDG. Multidirectional dynamics: decrease of metabolic activity in lumbar vertebral bodies (black arrows) and m. psoas on the right side (blue arrows) (**A**); appearance of metabolically active changes anterior to the descending thoracic (blue arrow) and abdominal aorta (black arrows) (**B**)**.**

A follow-up MRI and PET/CT with ^18^F-FDG showed mixed results: a regression of inflammatory changes in the retroperitoneal infiltrate and L2–L3 vertebrae but new lesions in the L4 vertebral body (Figure 3).

Antimicrobial therapy was subsequently adjusted to vancomycin 20 mg/kg administered intravenously every 12 h and moxifloxacin 400 mg delivered intravenously once daily. Despite this, PET/CT revealed additional lesions involving the Th10 vertebral body, intrathoracic, axillary, and retroperitoneal lymph nodes, spleen, and basal segments (S1–S2) of the left lung.

To exclude rare inflammatory pathogens, the patient underwent transesophageal puncture with an enzyme-linked immunosorbent assay (ELISA) to test for chronic brucellosis; the result was negative, thus excluding this diagnosis. Tuberculosis activity was assessed with Diaskintest® (Generium, Russia), culture, and T-SPOT, but all results were negative. In 4 June 2024, surgical removal of a right retroperitoneal mass was performed for the purpose of conducting a histological examination; this examination revealed granulomatous infiltration with caseous necrosis and multinucleated Pirogov–Langhans giant cells. However, the immunohistochemistry and PCR-RV of paraffin-embedded tissues did not detect *M. tuberculosis complex* DNA.

Despite the absence of direct microbiological confirmation, histological findings strongly suggested a tuberculous etiology. Therefore, antituberculosis chemotherapy was initiated with isoniazid (300 mg), rifampicin (450 mg), ethambutol (1,200 mg), and moxifloxacin (400 mg), which resulted in a positive clinical and laboratory response, including a decrease in CRP after 4 weeks.

### Surgical treatment

**Figure 4:**
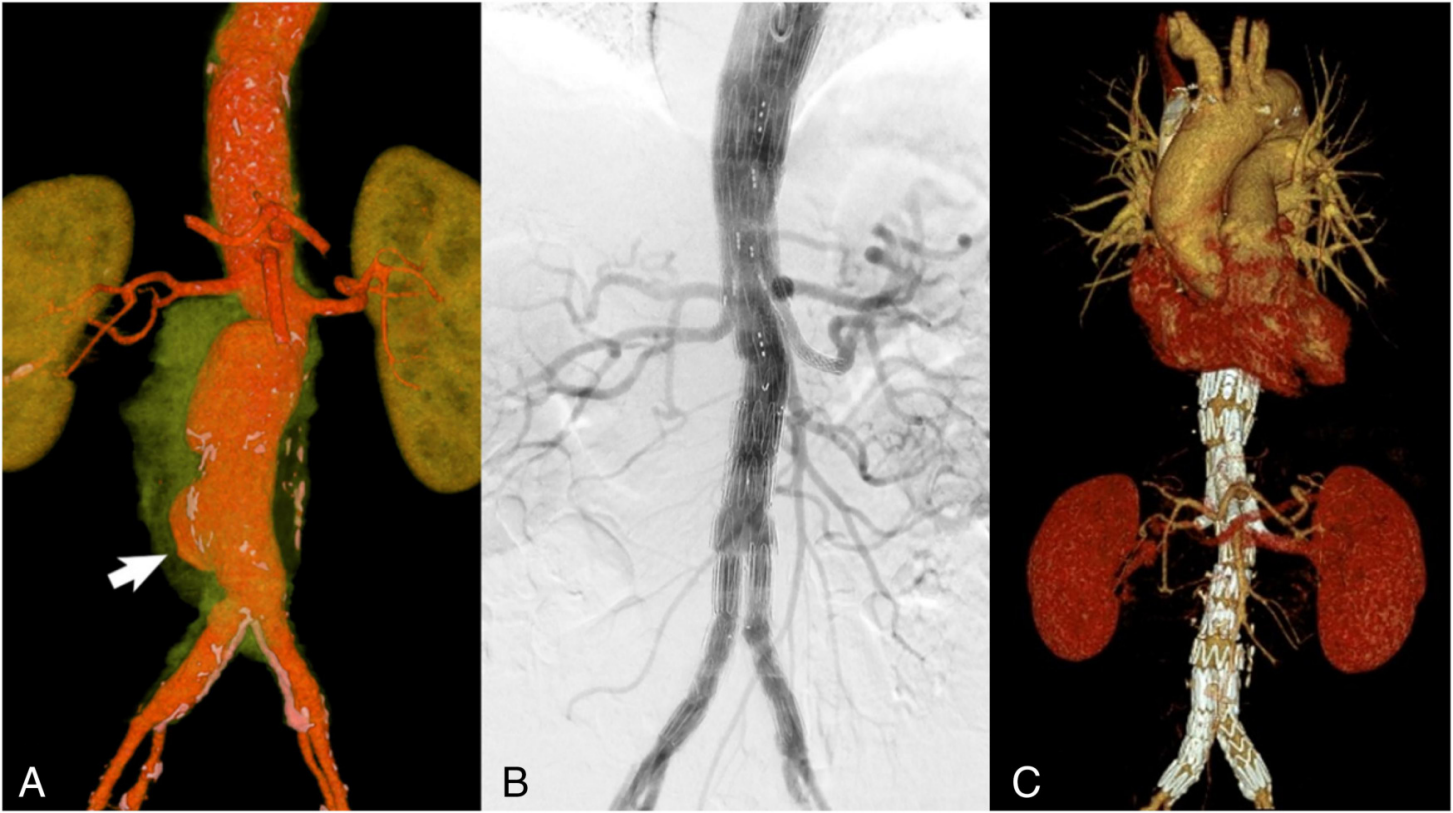
VRT reconstruction (spatial rendering) **(A)** CT scan with green-highlighted periaortic pararenal infiltration. The arrow indicates the area of the aortic wall defect and saccular aneurysm formation. Control angiography immediately after BEVAR with an endoprosthesis of all large visceral branches of the aorta **(B)** and 6 months after surgery **(C)**: all visceral arteries are patent with no signs of endoleaks.

A control CT angiography revealed an increase in the thoracoabdominal aorta diameter to 60 mm × 51 mm with a sac-like bulge at the bifurcation (Figure 4A). Given the high risk of rupture and considering the patient's advanced age and comorbidities, branched endovascular aortic repair (BEVAR) was selected as the treatment strategy.

The procedure was performed under general anesthesia with access gained through the left brachial and right common femoral arteries. A branched Zenith t-Branch stent graft (Cook Medical, Bloomington, Indiana, USA) was implanted, followed by selective catheterization and stent grafting of the renal arteries, the superior mesenteric artery (SMA), and the celiac trunk. The final angiography confirmed correct positioning of the device, absence of endoleaks, and preserved perfusion of all visceral arteries (Figure 4B).

### Outcomes

In the early postoperative period, the patient noted positive dynamics, specifically a reduction in pain syndrome severity. A postoperative CT angiography revealed a noticeable reduction in the size of the para-aortic hematoma and a diminished diameter of the infrarenal aorta. In July 2024, a control CT scan of the lumbosacral spine was performed, which demonstrated a stable radiological image characterized by bone atrophy with intact cortical **layers**, without an increase in the areas of vertebral destruction or evidence of sequestered fragments or abscesses. Thus, after repeated discussions, and also with neurosurgeons, it was decided that the patient did not need surgical stabilization of the spine.

Three weeks after discharge, the patient continued chemotherapy for at least 1 year according to the recommended schedule. Six months after surgery, a control CT angiography (Figure 4B) confirmed **patency of all visceral arteries**, including the renal arteries, the SMA, and the celiac trunk, with no evidence of endoleaks. Para-aortic tissues showed no changes, and areas of destruction in the L2–L4 vertebral bodies had decreased, displaying signs of progressive sclerosis along the margins.

## Discussion

Mycobacterial and tuberculosis infections are similar in clinical, radiological, and morphological manifestations. It is necessary to take into account that the spread of tuberculosis infection remains high in many countries worldwide.

According to the World Health Organization (WHO), an estimated 10.8 million TB cases were detected in 2023, up 3.5% from 10.7 million in 2022 and higher than the 10.4 million in 2021 and 10.1 million in 2020 (8). A study by Ohene et al. showed that out of 3,342 patients diagnosed with TB, 21.8% were diagnosed with extrapulmonary tuberculosis (EPTB) (9).

The first case of tuberculous aortitis was reported by Weigert in the year 1882 (10). Osler described a patient with multiple aneurysms secondary to mycotic endocarditis in 1885 (11). There are no precise statistics on the incidence of tuberculosis lesions of the aorta. According to studies, this localization of the tuberculosis process was diagnosed in 3.5% of patients with aortitis (12, 13).

The necessity of surgical intervention in aortic aneurysm with a tuberculosis lesion is obvious, but we should take into account the high percentage of lethal outcomes in urgent and open surgeries in 25.0% of patient cases. Lethal outcomes in patients with active tuberculosis and the presence of clinical symptoms reach 35%. Timely antituberculosis therapy before surgical intervention significantly reduces the number of complications and lethal outcomes (14). However, because of the increased risk of aneurysm rupture, patients with this pathology should undergo surgical treatment immediately after diagnosis, regardless of the aortic diameter (15). Infectious spondylitis occurring concurrently with a tuberculous aneurysm lesion is rare. According to the results of a retrospective analysis, a combination of mycotic aneurysm and patients with infectious spondylitis was diagnosed in 10.3% of cases (16).

Currently, tuberculous aortic aneurysm is a very rare pathology, which is attributed to early detection of tuberculosis in patients and the administration of antituberculosis chemotherapy. Most tuberculous aneurysms are false [approximately 88% (17)], i.e., they are caused by focal infiltration of the vessel wall with destruction of all its layers (6), rupture of the aneurysm, and its restriction by surrounding tissues.

Virtually all aortic segments can be involved, but the most frequently affected segments are those in close proximity to mediastinal and para-aortic lymph nodes. The clinical picture in tuberculous aortic aneurysm varies widely from an asymptomatic course to a pronounced symptomatology, especially in case of complications. In addition to non-specific symptoms (fever and weight loss), the main clinical manifestations include the following: signs of palpable pulsating mass in the abdomen; chest pain, dysphagia, and hoarseness of voice in thoracic aortic lesions; and abdominal or back pain in abdominal aortic lesions. Possible complications include fistula formation, aortic perforation or rupture with subsequent hemorrhage, and hemorrhagic shock (18).

Rupture of a tuberculous aortic aneurysm is a life-threatening condition requiring immediate surgical intervention, which includes open aortic repair or endovascular aortic repair (EVAR). In case of a favorable anatomy in the rupture of mycotic aneurysms of the thoracoabdominal aorta in elderly comorbid patients, endovascular intervention (use of fenestrated/branched stent graft) is preferable due to its lower invasiveness and acceptable results on early postoperative morbidity and mortality in these patients (13, 17, 19). Along with surgical treatment of this category of patients, chemotherapy is mandatory (20, 21).

Our observations are in agreement with the data of foreign literature. Pluemvitayaporn et al. (22) presented a clinical case that described a patient with a ruptured tuberculous SMA with concomitant tuberculous spondylitis at the level of the L3, L4 vertebrae, left-sided psoas abscess, and cauda equina syndrome. The patient underwent retroperitoneal removal and EVAR, followed by a decompression laminectomy of the L3, L4 vertebrae and transpedicular fixation of the Th11-L5 vertebrae.

After these interventions, the patient showed a regression of neurological symptoms. Zhang et al. (23) reported the case of a patient with a tuberculous pseudoaneurysm of the abdominal aorta with concomitant tuberculous lesions of the kidney and L3–L5 vertebrae, as well as left-sided psoas abscess; the patient underwent EVAR, followed by a surgical stabilization of the lumbar spine.

Chen et al. (24) described a patient with a tuberculous pseudoaneurysm of the thoracic aorta with concomitant tuberculous lesions of the Th11-L1 vertebrae and bilateral paravertebral cold abscess. The patient underwent abscess drainage as well as thoracic EVAR and spondylodesis with internal fixation of the vertebrae. In the early postoperative period, the patient had a regression of neurological symptoms. After 3 months, a CT angiography showed a passable reconstruction zone and no endoleaks and/or stent graft migration. The early postoperative period proceeded without the patient experiencing any complications. Five months after surgery, another CT angiography showed a passable reconstruction zone and a decrease in inflammatory phenomena in the retroperitoneal space.

Obviously, against the background of the use of a large number of instrumental methods, immunological diagnostic methods, in combination with morphological and molecular genetic methods, can be useful in making a diagnosis (25, 26). The possibilities for conducting new immunological tests, including the Russian skin test (Diaskintest), have made it possible to increase the level of diagnostics of tuberculosis infection. In the Russian Federation, the test is actively used in clinical practice, including for making a differential diagnosis of tuberculosis with other granulomatous diseases (27, 28).

However, the possibility for performing negative immunological tests in patients with extrapulmonary localizations of tuberculosis should be considered. In the case of our patient, negative results of immunologic tests were obtained. In extrapulmonary localizations of TB, false-negative QuantiFERONTB®-Gold (QFT-G) test results were reported in 28.8% of cases. Among TB patients with bone and joint TB, negative QFT-G test results were detected in 46.2% (29). It is necessary to obtain bacteriological and morphological examination data, which, however, is not always possible.

The clinical case described by us is of particular interest because of the absence of vivid clinical symptoms in a patient with a ruptured thoracoabdominal aortic aneurysm. At the same time, the polymorphism of pathological changes detected by CT angiography (TAA, destruction of vertebral bodies, and abscess of m. psoas major) attracted attention. The only manifestation of this condition was a pronounced pain syndrome in the lumbar region, which, in turn, made the diagnosis difficult. This fact dictates the necessity of early detection of aortic aneurysm in patients with tuberculosis and the fastest possible initiation of active treatment to prevent the development of life-threatening complications.

The few publications describing the possibilities of surgical treatment of mycobacterial lesions of the aorta confirm the need for adjuvant antimycobacterial therapy, which should be initiated immediately after diagnosis (7, 30).

Despite the inherent risks of infectious complications, endovascular procedures demonstrate significant advantages over open surgery because they minimize the need for extensive tissue incisions and allow avoiding prolonged hospital stays. This reduces the risk of patients acquiring hospital-acquired infections and shortens the recovery period. Numerous publications provide ample evidence of the effectiveness of endovascular methods even in complex clinical scenarios, thereby justifying their recommendation for patients at high risk of infection (31–34).

The description of endovascular treatment of multiple mycotic aortic aneurysms caused by tuberculosis using micro-stent grafts is extremely rare. Nevertheless, some publications emphasize that endovascular intervention improves hemodynamics and eliminates the risk of aneurysm rupture. Special emphasis is placed on combining endovascular treatment with antituberculosis therapy (35). A retrospective analysis of the results of studying the effectiveness of EVAR in patients with tuberculous aneurysms demonstrated the effectiveness and durability of EVAR in combination with antituberculosis therapy. The literature reports a clinical case of endovascular treatment of tuberculous pseudoaneurysm of the abdominal aorta. Endovascular intervention (EVAR) is presented as a successful method of treatment within a comprehensive approach (36).

## Conclusion

The presented clinical case demonstrates the possibility of radial diagnostic methods in detecting pathological changes in the thoracoabdominal aorta and adjacent organs, as well as the possibility of endovascular treatment of a patient with a ruptured mycobacterial aneurysm of the thoracoabdominal aorta with secondary spondylitis and psoas abscess. This technique seems to us an acceptable alternative to open aortic prosthesis in elderly patients with a severe comorbid background, but further clinical studies are needed to confirm this assumption. Vascular lesions with fast-growing mycobacteria and mycobacteria of the tuberculosis complex are rarely encountered in clinical practice. However, because of the prevalence of cardiovascular devices in older patients and in a setting of spreading tuberculous infection, the need for timely and optimized treatment of these infections is of great importance.

## Data Availability

The raw data supporting the conclusions of this article will be made available by the authors without undue reservation.
