# Supplementary figures and images for: Case Report: Endovascular repair of thoracoabdominal aneurysm aorta and generalized mycobacterial infection (clinical case)
Source: Front Cardiovasc Med. 2025 Nov 6;12:1623403. doi: 10.3389/fcvm.2025.1623403 (PMC12631293; doi:10.3389/fcvm.2025.1623403)

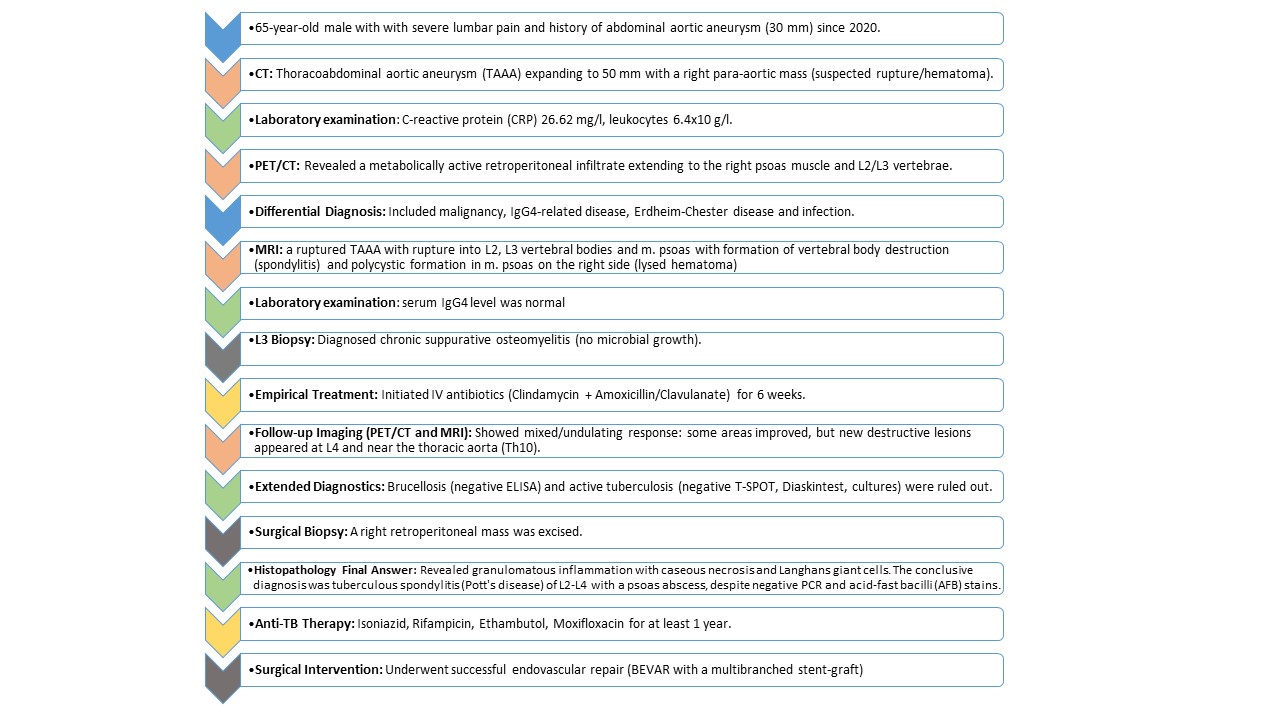

Supplement: Supplementary file 1 [file Image1.jpeg]
